# O Escore de Risco GRACE é o Santo Graal na Estratificação de Risco ou Podemos Melhorá-lo ainda mais com Biomarcadores Adicionais?

**DOI:** 10.36660/abc.20200171

**Published:** 2020-05-12

**Authors:** Ana Teresa Timóteo

**Affiliations:** Hospital Santa Marta Centro Hospitalar Universitário Lisboa Central Lisboa Portugal Serviço de Cardiologia - Hospital Santa Marta - Centro Hospitalar Universitário Lisboa Central, Lisboa – Portugal

**Keywords:** Síndrome Coronariana Aguda, Insuficiência Cardíaca, Peptídeo Natriurético, Fator Natriurético Atrial, Mortalidade, Disfunção Ventricular Esquerda, Biomarcadores, Encéfalo

O peptídeo natriurético do tipo B (BNP) tem sido reconhecido como um marcador muito útil para a detecção de disfunção ventricular esquerda aguda e crônica, ambas sistólica e diastólica, que pode estar presente no contexto de isquemia miocárdica súbita e prolongada.^1 , 2^ Estes são os primeiros passos da cascata isquêmica, levando à necrose celular. Por esse motivo, os peptídeos natriuréticos geralmente estão elevados no contexto de síndromes coronárias agudas.^2^

A isquemia miocárdica, mesmo na ausência de disfunção ventricular esquerda, aumenta a expressão do gene do BNP cardíaco, aumentando as concentrações plasmáticas de NT-proBNP.^3 , 4^ A cinética do BNP geralmente atinge o pico após 16 horas do início dos sintomas no infarto do miocárdio, com supradesnivelamento do segmento ST e um segundo pico é geralmente observado no quinto dia.^5^ Podemos especular que o primeiro pico possa estar associado à isquemia e o segundo pico à disfunção ventricular esquerda associada à necrose celular e remodelação precoce.

A porção N-terminal do pró-hormônio do peptídeo natriurético do tipo B (NT-proBNP) é o produto amino-terminal após a clivagem do peptídeo precursor do BNP. Possui meia-vida mais longa, permitindo maior acúmulo e sensibilidade na detecção de alterações estruturais e funcionais sutis.^5 , 6^ O NT-proBNP foi extensivamente estudado nas últimas duas décadas, principalmente nos anos 2000, e os resultados mostraram consistentemente que medidas iniciais fornecem informações importantes e independentes para estratificação de risco em todo o espectro de síndromes coronarianas agudas.^7 - 10^ A precisão prognóstica das medidas iniciais de NT-proBNP é ainda melhor quando comparada às medidas iniciais de troponina cardíaca, refletindo o insulto isquêmico em vez da necrose celular.^9^

O escore de risco GRACE é atualmente o escore de estratificação de risco mais amplamente recomendado no contexto de síndromes coronárias agudas.^11 , 12^ Ele incorpora marcadores clínicos, de eletrocardiograma e bioquímicos e é altamente preditivo de mortalidade em curto e médio prazo. Para a mortalidade intra-hospitalar, geralmente são obtidos valores de Área Sob a Curva (AUC, do inglês *Area Under the Curve* )> 0,85.^7^ Entretanto, estudos anteriores não demonstraram nenhum benefício adicional com a inclusão de peptídeos natriuréticos nesta ferramenta de estratificação de risco.^7^

O artigo de Souza et al.,^13^ estudou o valor preditivo independente do NT-pro-BNP comparado à classe Killip-Kimball em pacientes com todo o espectro de síndromes coronárias agudas e o valor incremental potencial quando incluídos no escore de risco GRACE em substituição à classe Killip.^13^ Eles estudaram 352 pacientes com uma média de idade de 63 anos, 60% do sexo masculino, 26% com infarto do miocárdio com supradesnivelamento do segmento ST e mortalidade cardiovascular hospitalar de 4,8%. O NT-pro-BNP foi medido na hospitlização, em uma mediana de 15,5 horas após o início dos sintomas e 29% apresentaram níveis aumentados. O NT-pro-BNP mostrou uma precisão preditiva moderada com uma AUC de 0,78, melhor do que a classe Killip. No entanto, não foi superior em comparação ao escore de risco GRACE tradicional (AUC 0,82) ou quando incluído no escore GRACE (AUC 0,83). Também não houve benefício em termos de análise de reclassificação.

Os resultados apresentados estão alinhados com estudos anteriores, confirmando, em uma coorte contemporânea de pacientes, o valor prognóstico independente na hospitalização de NT-proBNP nas síndromes coronárias agudas e os resultados da AUC também foram semelhantes. A principal originalidade do presente trabalho é o uso desse biomarcador não como um complemento, mas em substituição à classe Killip, um dos marcadores clínicos do escore de risco GRACE, justificado pela colinearidade esperada entre a classe Killip e o NT-proBNP. Entretanto, mesmo com essa abordagem, o NT-proBNP não melhorou a precisão prognóstica do escore de risco GRACE. Acredito que a principal explicação é que o escore de risco GRACE é um escore tão potente, com uma AUC geralmente relatada como >0,85, incluindo variáveis prognósticas já muito importantes, que é muito difícil melhorar ainda mais essa precisão prognóstica. Vários outros marcadores foram testados por outros autores e resultados semelhantes foram obtidos, sem nenhuma melhora significativa. Os resultados poderiam ser diferentes no seguimento em longo prazo? Essa é uma pergunta importante que pode ser respondida em estudos subsequentes.

Há também algumas limitações adicionais no presente estudo. A inclusão foi realizada por seis anos, mas apenas 352 pacientes foram incluídos. Embora a amostra seja adequada em relação ao tamanho da amostra do estudo apresentado, ela sugere que a inclusão não foi consecutiva e vários pacientes não foram considerados. Essa é uma fonte potencial de viés. Outro fato importante é que nenhum dado é apresentado sobre características basais importantes. Por essa razão, não podemos avaliar se a amostra realmente representa as características usuais do paciente nas coortes de síndromes coronárias agudas. Também não sabemos quais foram os ajustes feitos na análise multivariada. Ou seja, se todas as variáveis com possível impacto no prognóstico e nos níveis de NT-proBNP foram consideradas no ajuste multivariado. A definição de “insuficiência cardíaca” utilizada pelos autores também não está claramente explicada.

Em conclusão, o presente estudo mostra que em uma coorte contemporânea de pacientes com todo o espectro de síndromes coronarianas agudas, embora o NT-proBNP tenha um valor prognóstico moderado independente para a mortalidade cardiovascular hospitalar, ele não melhora a precisão prognóstica da estratificação de risco do escore de risco GRACE. Mas precisamos aprimorá-lo e adicionar complexidade substancial ao seu uso? Não acredito que seja esse o caso.
