# Supplementary material for: O Escore de Risco GRACE é o Santo Graal na Estratificação de Risco ou Podemos Melhorá-lo ainda mais com Biomarcadores Adicionais?
Source: Arq Bras Cardiol. 2020 May 12;114(4):673–4. [Article in Portuguese] doi: 10.36660/abc.20200171 (PMC9744346; doi:10.36660/abc.20200171)
Supplement: Translation

# Translation: Is Grace Risk Score the Holy Grail in Risk Stratification or Can We Improve it Even Further with Additional Biomarkers?

**Authors:** Ana Teresa Timóteo

**Affiliations:** Portugal Serviço de Cardiologia - Hospital Santa Marta - Centro Hospitalar Universitário Lisboa Central, Lisboa – Portugal

**Keywords:** Acute Coronary Syndrome, Heart Failure, Natriuretic Peptide, Factor, Natriuretic Atrial, Mortality, Ventricular Dysfunction, Left, Biomarkers, Brain

B-type natriuretic peptide (BNP) has been recognized as a very useful marker for the detection of acute and chronic left ventricular dysfunction, both systolic and diastolic, that can be present in the context of sudden and prolonged myocardial ischemia.^1 , 2^ These are the first steps in the ischemic cascade, leading to cell necrosis. For that reason, natriuretic peptides are usually elevated in the context of acute coronary syndromes.^2^

Myocardial ischemia, even in the absence of left ventricular dysfunction, augments cardiac BNP gene expression, increasing plasma NT-proBNP concentrations.^3 , 4^ BNP kinetics usually peaks at 16 hours of symptom’s onset in ST-elevation myocardial infarction and a second peak is usually observed by the fifth day.^5^ We can speculate that the first peak might be associated with ischemia and the second peak to left ventricular dysfunction associated with cell necrosis and early remodelling.

N-terminal-pro-BNP (NT-proBNP) is the amino-terminal product after cleavage of the precursor peptide of BNP. It has a longer half-life, allowing greater accumulation and sensitivity in detecting subtle structural and functional changes.^5 , 6^ NT-proBNP has been extensively studied in the last two decades, particularly in the 00’s, and results consistently showed that early measurements provide important and independent information for risk stratification across the entire spectrum of acute coronary syndromes.^7 - 10^ Prognostic accuracy of early NT-proBNP measurements is even better when compared to early cardiac troponin measurements, reflecting the ischemic insult rather than cell necrosis.^9^

GRACE risk score is currently the most widely recommended risk stratification score in the context of acute coronary syndromes.^11 , 12^ It incorporates clinical, electrocardiogram and biochemical markers and it is highly predictive for short- and medium-term mortality. For in-hospital mortality, values of Area Under Curve > 0.85 are usually obtained.^7^ However, previous studies did not show any additional benefit with the inclusion of natriuretic peptides in this risk stratification tool.^7^

The article by Souza et al.^13^ studied the independent predictive value of NT-pro-BNP compared to Killip-Kimbal class in patients with the whole spectrum of acute coronary syndromes and the potential incremental value when included in GRACE risk score in substitution of Killip class.^13^ They studied 352 patients with a mean age of 63 years, 60% males, 26% with ST-elevation myocardial infarction and in-hospital cardiovascular mortality was 4.8%. NT-pro-BNP was measured on admission, at a median of 15.5 hours after symptoms onset and 29% showed increased levels. NT-pro-BNP showed a moderate predictive accuracy with an AUC of 0.78, better than Killip class. However, it was not superior compared to the traditional GRACE risk score (AUC 0.82) or when included in GRACE score (AUC 0.83). There was also no benefit in terms of reclassification analysis.

The results presented are in line with previous studies, confirming, in a contemporaneous cohort of patients, the independent prognostic value of admission NT-proBNP in acute coronary syndromes and AUC results were also similar. The main originality of the present paper is the use of this biomarker not as an add-on but in substitution for Killip class, one of the clinical markers of GRACE risk score, justified by the collinearity expected between Killip class and NT-proBNP. However, even with this approach, NT-proBNP didn’t improve the prognostic accuracy of the GRACE risk score. I believe that the main explanation is that GRACE risk score is such a potent score, with an AUC usually reported as > 0.85, including already very important prognostic variables, that is very difficult to improve even further this prognostic accuracy. Several other markers were tested by other authors and similar results of no significant improvements were obtained. Could the results be different in long-term follow-up? This is an important question that can be answered in subsequent studies.

There are also some additional limitations to the present study. Inclusion was performed for six years, but only 352 patients were included. Albeit the sample is adequate according to the sample size study presented, it suggests that the inclusion was not consecutive, and several patients were not considered. This is a potential source of bias. Another important fact is that no data is presented about important baseline characteristics. For that reason, we cannot assess if the sample really represents the usual patient’s characteristics in acute coronary syndromes cohorts. We also do not know what were the adjustments made in multivariate analysis. That is if all variables with a possible impact in prognosis and in NT-proBNP levels were considered in the multivariate adjustment. The “heart failure” definition used by the authors is also not clearly explained.

In conclusion, the present study shows that in a contemporaneous cohort of patients with the whole spectrum of acute coronary syndromes, although NT-proBNP has an independent moderate prognostic value for in-hospital cardiovascular mortality, it does not improve the risk stratification prognostic accuracy of the GRACE risk score. But do we need to improve it and add substantial complexity to its use? I do not believe that this is the case.
